# Nitric oxide interacts with cholinoceptors to modulate insulin secretion by pancreatic β cells

**DOI:** 10.1007/s00424-020-02443-9

**Published:** 2020-08-16

**Authors:** Bashair M. Mussa, Ankita Srivastava, Abdul Khader Mohammed, Anthony J. M. Verberne

**Affiliations:** 1 Basic Medical Science Department, College of Medicine, University of Sharjah, P.O. Box: 27272, Sharjah, United Arab Emirates; 2 Sharjah Institute for Medical Research, University of Sharjah, P.O. Box: 27272, Sharjah, United Arab Emirates; 3 Department of Medicine, Austin Health, University of Melbourne, Heidelberg, Victoria 3084 Australia

**Keywords:** Cytokines, Nitric oxide, Cholinoceptors, Insulin secretion, Pancreatic β cells

## Abstract

Dysfunction of the pancreatic β cells leads to several chronic disorders including diabetes mellitus. Several mediators and mechanisms are known to be involved in the regulation of β cell secretory function. In this study, we propose that cytokine-induced nitric oxide (NO) production interacts with cholinergic mechanisms to modulate insulin secretion from pancreatic β cells. Using a rat insulinoma cell line INS-1, we demonstrated that β cell viability decreases significantly in the presence of SNAP (NO donor) in a concentration- and time-dependent manner. Cell viability was also found to be decreased in the presence of a combined treatment of SNAP with SMN (muscarinic receptor antagonist). We then investigated the impact of these findings on insulin secretion and found a significant reduction in glucose uptake by INS-1 cells in the presence of SNAP and SMN as compared with control. Nitric oxide synthase 3 gene expression was found to be significantly reduced in response to combined treatment with SNAP and SMN suggesting an interaction between the cholinergic and nitrergic systems. The analysis of gene and protein expression further pin-pointed the involvement of M_3_ muscarinic receptors in the cholinergic pathway. Upon treatment with cytokines, reduced cell viability was observed in the presence of TNF-α and IFN-γ. A significant reduction in insulin secretion was also noted after treatment with TNF-α and IFN-γ and IL1-β. The findings of the present study have shown for the first time that the inhibition of the excitatory effects of cholinergic pathways on glucose-induced insulin secretion may cause β cell injury and dysfunction of insulin secretion in response to cytokine-induced NO production.

## Introduction

Pathophysiology of diabetes mellitus (DM) is highly complex, and multiple factors and pathways are involved. β cell dysfunction is considered to be the main pathological feature of DM, and previous studies have identified several mediators of β cell injury that are associated with DM. However, the mechanisms by which these mediators influence insulin secretion are yet to be identified [10, 29, 41].

For decades, type 1 DM was considered as the sole type of DM that is associated with β cell destruction and subsequent reduction in insulin secretion, whereas type 2 DM was mainly characterized by increased insulin resistance. Nevertheless, several lines of recent evidence have confirmed that β cell loss also plays an important role in the pathogenesis of type 2 DM in the long term. β cell failure and disturbance of insulin secretion affects many organs and leads to dramatic complications including cardiovascular disease, stroke, renal failure, and neuropathy [8, 34, 44].

It is noteworthy that the mechanisms that underpin β cell failure in the progression of type 1 and type 2 DM differ considerably. In the former, the main process that leads to destruction of the β cell is based on autoimmune-mediated apoptosis, whereas in type 2 DM, the dysfunction of β cells mainly involves circulating cytokines (interleukin 1 beta, IL-1β; tumor necrosis factor alpha, TNF-α; and interferon-γ, IFN-γ) which indirectly promote apoptosis through excessive production of nitric oxide (NO) [39]. NO is a free radical molecule which has several physiological and pathological functions. It is generated by the oxidation of the amino acid l-arginine by a family of enzymes known as nitric oxide synthases (NOS). Three distinct isoforms of NOS are known: (i) two isoforms that are constitutively expressed in neurons (nNOS), (ii) endothelial (eNOS) cells, as well as (iii) an inducible isoform (iNOS) which is expressed primarily by immune cells (e.g., macrophages) [8, 34]. The constitutively expressed isoforms release low levels of NO that exert physiological functions, whereas iNOS releases a high output of NO production in response to immunogenic and inflammatory stimuli [38, 43].

It has been strongly suggested that NO mediates the inhibitory actions of cytokines on β cell function by inhibiting glucose oxidation to CO_2_ as well as by reducing cellular levels of ATP [38, 42]. Although a large amount of research has been conducted to identify the elements that are involved in β cell injury, there is still a debate about the effects of NO in reducing β cell mass and insulin secretion.

Interestingly, our previous findings have speculated that an interaction between NO and cholinergic mediators influences insulin secretion [24]. We have identified and characterized the central pathways that are involved in the control of insulin secretion in vivo [24]. The main site of origin of all vagal efferent neurons that innervate the endocrine pancreas is the dorsal motor nucleus of the vagus (DMV) [25]. DMV vagal efferent neurons synapse onto cholinergic and non-cholinergic neurons in the pancreas, and it has been demonstrated that these neurons directly influence glucose-induced insulin secretion [24, 40]. It is well-documented that electrical and chemical stimulations of the DMV result in a significant increase in glucose-induced insulin secretion [25]. These excitatory effects on glucose-induced insulin secretion were completely inhibited by peripheral blockade of muscarinic receptors confirming that a cholinergic pathway plays a major role in enhancing glucose-induced insulin secretion [24]. On the other hand, it has been demonstrated that the blockade of peripheral NO synthase potentiated the excitatory effects of cholinergic pathway activation on glucose-induced insulin secretion [24]. This finding strongly suggests an interaction between acetylcholine (ACh), the main neurotransmitter of the peripheral cholinergic pathways, and NO to regulate glucose-induced insulin secretion. In addition, in vivo and in vitro studies have supported this hypothesis by emphasizing the critical role of ACh in maintaining glucose homeostasis and by demonstrating the presence of muscarinic ACh receptors on the β cell [1, 13]. Therefore, our present hypothesis is that cytokine-stimulated NO interacts with cholinergic pathways to modulate insulin secretion from β cells.

Taking all these previous findings into account, the present study was designed to investigate the involvement of NO and muscarinic receptors on survival of β cells and the regulation of glucose-induced insulin secretion.

## Materials and methods

### Cell culture and viability

Rat insulinoma cell line INS-1 832/13, a subclone producing both rat and human insulin, transfected with a CMV promoter carrying a neomycin resistance marker was obtained from Lund University, Sweden, by Dr. Jalal Taneera and was used as our research model. INS-1 832/13 pancreatic cells have been used widely in the field of insulin secretion research. These cells were cultured in RPMI 1640 supplemented with 10% heat inactivated FBS (fetal bovine serum), 100 IU/ml penicillin, 100 μg/ml streptomycin, 10 mM HEPES (4-(2-hydroxyethyl)-1-piperazineethanesulfonic acid), 2 mM l-glutamine, 1 mM sodium pyruvate, and 50 μM β-mercaptoethanol [17].

Measurement of cell viability was as follows: 1 × 10^4^ cells were seeded per well in 96-well plates (Jet Biofil, Guangzhou, China) with 200 μl RPMI 1640 culture medium and maintained in the incubator at 37 °C with 5% CO_2_. Upon reaching > 90% confluency, cells were treated in triplicates with 50 μM, 100 μM, and 200 μM of SNAP (*S*-nitroso-*N*-acetylpenicillamine) or LNNA (L-NG-nitro-l-arginine) along with their vehicles 200 μM dimethyl sulfoxide (DMSO) and 200 μM hydrochloric acid (HCl), respectively. Cell viability was also measured in the presence of scopolamine methyl (SMN) at a concentration of 0.2 nM.

Viability of the cells in the presence of cytokines was measured as follows: 2 × 10^4^ cells were seeded in 0.1 ml/well culture media in 96-well plate and allowed to adhere overnight. Cells were then incubated for 24 h in the presence of pro-inflammatory cytokines: 125 ng/ml of tumor necrosis factor-alpha (TNF-α; Abcam9756), 100 ng/ml of interleukin 1-beta (IL1-β; Abcam9788), and 125 ng/ml of interferon-gamma (IFN-γ; Abcam645). Cells without any treatment were regarded as controls. After treatment, viability was checked using a colorimetric assay with the tetrazolium dye MTT (3-[4,5-dimethylthiazol-2-yl]-2,5-diphenyltetrazolium bromide). Culture medium was discarded and replaced with a mixture containing 20 μl of MTT (5 mg/ml) dissolved in 100 μl of PBS (phosphate-buffered saline). Cells were then incubated at 37 °C for 2 h in the dark. Post incubation, 100 μl of DMSO was added to each well to dissolve the formazan crystals formed by MTT and absorbance was recorded at 570 nm on a microplate reader with 630 nm wavelength as reference. The percentage of cell viability was calculated from the average 570 nm absorbance value as per the following equation: % cell viability = (OD of sample at 570 nm/OD of control at 570 nm) × 100.

### Nitrite measurement

The concentration of nitrite released by the cells was measured using 50 μl of spent media which was collected from each well after 24 h of treatment with the cytokines—TNF-α (125 ng/ml), IL1-β (10 ng/ml), and IFN-γ (125 ng/ml), respectively. Nitrite concentration was measured using the Greiss Reagent System (Promega Corporation, Madison, USA) as per the manufacturer’s protocol. The absorbance was recorded at 595 nm in a microplate reader using 630 nm wavelength as reference. The concentration of nitrite in each sample was determined using a standard reference curve which was generated to compare the average absorbance value for each sample.

### Insulin secretion assay

Briefly, cells were seeded at density of 3 × 10^5^ in 1 ml/well culture media in a 24-well plate (Jet Biofil, Guangzhou, China) and were allowed to adhere overnight. Cells were then exposed to cytokines—IL-1β (100 ng/ml), TNF-α (125 ng/ml), IFNγ (125 ng/ml), or the nitric oxide donor SNAP (100 μM and 200 μM) or muscarinic receptor antagonist (SMN 0.2 nM) for 24 h. The cells were then washed twice with 1 ml of pre-warmed secretion assay buffer (SAB), pH 7.2 (114 mM NaCl, 4.7 mM KCl, 1.2 mM KH_2_ PO_4_, 1.16 mM MgSO_4_, 20 mM HEPES, 2.5 mM CaCl_2_, 25.5 mM NaHCO_3_, and 0.2% bovine serum albumin) containing 2.8 mM glucose. The cells were then incubated in 2 ml SAB containing 2.8 mM glucose for 2 h. Subsequently, cells were incubated for 1 h in 1 ml SAB containing either 2.8 mM or 16.7 mM glucose. The supernatants were collected and secreted insulin was measured using rat insulin ELISA kit (Elabscience, Wuhan, China) according to the manufacturer’s instructions. Secreted insulin was normalized to the protein content as determined using Bradford Assay (Bio-Rad Laboratories, Hercules, USA) [4].

### Gene expression

INS-1 cells were harvested for the isolation of RNA using the PureLink RNA Mini Kit (Invitrogen, Carlsbad, USA). RNA was isolated following the manufacturer’s protocol with final elution volume of 30 μl. The isolated RNA was then quantified using the Nanodrop2000 spectrophotometer (Thermo Fisher Scientific, Waltham, USA), and purity was determined by the A260/A280 ratio. From this, 0.5 μg RNA was used to reverse transcribe to cDNA with 10 mM dNTP mix and 5 μM random primers (high-capacity cDNA synthesis kit; Applied Biosystems, Foster City, USA) and incubated at 70 °C for 10 min, followed by addition of 200 units of M-MLV reverse transcriptase in a reverse transcriptase buffer and incubated at 37 °C for 50 min and 90 °C for 10 min in a Veriti thermal cycler (Applied Biosystems, Foster City, USA). The final reaction volume was 20 μl.

The analysis of gene expression was checked, and quantitative real-time PCR was performed using the StepOne Real Time PCR system (Applied Biosystems, Foster City, USA) in a total reaction volume of 10 μl containing 5 μl of 1× Power SYBR green master mix (Applied Biosystems, Foster City, USA), 0.5 μl of 10 μM primers (Table 1), and 0.5 μg of cDNA. The cycling parameters included initialization at 95 °C for 2 min followed by denaturation at 95 °C for 15 s and annealing/extension at 62 °C for 1 min for a total of 40 cycles. Purity of the obtained PCR products was confirmed with a melt curve involving sequential heating at 95 °C for 15 s, 62 °C for 1 min, and 95 °C for 15 s. The rat primer sequences used for specific amplification with nitric oxide synthase 3 (NOS_3_), muscarinic acetylcholine receptor 3 (M_3_), and muscarinic acetylcholine receptor 5 (M_5_) are listed in Table 1. Relative gene expression was determined using the 2^ΔΔCt^ method, and rat hypoxanthine guanine phosphoribosyltransferase (HPRT) was used as the house keeping gene. Samples implemented with nuclease free water instead of cDNA were regarded as negative controls. All primer sequences were synthesized using the Primer3 software.

**Table 1:** Rat primers used for quantitative real-time polymerase chain reaction

Gene	Forward primer (5′–3′)	Annealing temp. (°C)	Reverse primer (3′–5′)	Annealing temp. (°C)	Product size (bp)
HPRT	GCAGACTTTGCTTTCCTTGG	53.4	TCCACTTTCGCTGATGACAC	53.4	157
NOS_3_	GATGGCGAAGCGTGTGAAGG	57.5	GGCCTCATGCTCTAGGGATACC	60.9	208
M3	TGCGCAGACAAGACCACGGC	62	GCGTCTGGGCGGCCTTCTTC	62	142
M5	TTGCAGTTGTGACTGCGGTG	56	GAGAACCCAGCGTCCCATGA	57	197

### Protein purification and analysis using western blotting

The following protocol was used for protein analysis: 1.5 × 10^6^ cells were seeded in 100 mm^2^ cell culture dish (Thermo Fisher Scientific, Waltham, USA) and grown at 37 °C with 5% CO_2_ till > 90% confluency was reached. Cells were then treated with the desired concentrations of SNAP (50 μM, 100 μM, and 200 μM) and SMN (0.2 nM) depending on the experiments. After 24 h of treatment, cells were harvested for protein extraction using M-PER mammalian protein lysis buffer (Thermo Fisher Scientific, Waltham, USA) containing a protease inhibitor cocktail (Thermo Fisher Scientific, Waltham, USA) in a 1:1000 dilution. The concentration of total protein was determined using the Bradford reagent (Bio-Rad Laboratories, Hercules, USA) and quantified by the Bradford Method [16]. Fifty micrograms of total protein from each sample was separated using 12% sodium dodecyl sulfate–polyacrylamide gel electrophoresis (SDS-PAGE) and transferred onto nitrocellulose membranes (Bio-Rad Laboratories, Hercules, USA) for 30 min at 20 V in a transfer buffer containing Tris base, methanol, and glycine. The membranes were blocked with 5% skim milk powder prepared in Tris-buffered saline with 0.1% of Tween 20 (TBST) for 1 h at room temperature and then incubated overnight at 4 °C with primary antibodies against M_3_ (1:1000 dilution; Abcam87199; Abcam, Cambridge, UK), M_5_ (1:750 dilution; Abcam41171; Abcam, Cambridge, UK), and β-actin (1:5000 dilution). The membranes were washed with TBST and incubated with horseradish peroxidase–linked secondary antibody (1:1000 dilution; Cell Signaling Technology, Danvers, USA) at room temperature for 1 h. After incubation, the membranes were washed with TBST and visualized using clarity western ECL substrate (Bio-Rad Laboratories, Hercules, USA) according to the manufacturer’s protocol. The intensity of the observed bands was quantified using ImageJ software.

All reagents and solutions were obtained from Sigma-Aldrich (St. Louis, USA) unless mentioned otherwise.

### Statistical analysis

All data are expressed as mean ± standard error, and differences between individual means were assessed using one-way analysis of variance (ANOVA) followed by the Tukey multiple comparison tests. Probability values of *P* < 0.05 were considered as statistically significant.

## Results

### Effects of NO donors and inhibitors on the survival rate of the INS-1 pancreatic cells

**Fig. 1:**
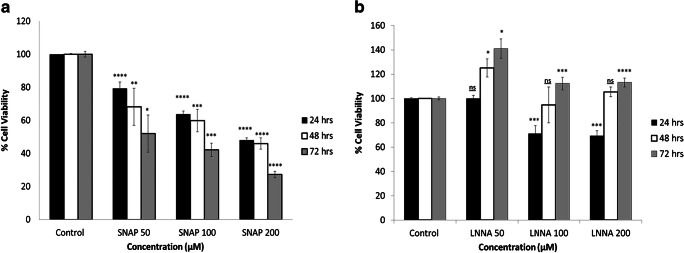
Effects of SNAP and LNNA on the viability of INS-1 pancreatic cells. **a** SNAP produced a concentration-dependent reduction in the survival rate of the INS-1 pancreatic cells. As the time of the exposure increased from 24 to 72 h, fewer cells survived (*n* = 3, *P < 0.05, **P < 0.01, ***P < 0.001, ****P < 0.0001, *P* values refer to the differences in concentrations compared with control conditions). **b** The presence of LNNA produced differential effects on the survival of the INS-1 pancreatic cells. Short exposure to two different concentrations of LNNA (100 and 200 μM) produced a reduction in the survival of the cells but this pattern was reversed as the time of exposure increased. Exposure for 72 h of LNNA (100 and 200 μM) increased the survival of the of the INS-1 pancreatic cells (*n* = 3, *P < 0.05, ***P < 0.001, ****P < 0.0001, *P* values refer to the differences in concentrations compared with control conditions). All values are expressed as ± SE. Abbreviations: INS-1, insulin-producing pancreatic cells; LNNA, L-NG-nitro-l-arginine; NS, not significant; SNAP, *S*-nitroso-*N*-acetylpenicillamine

Two separate sets of experiments were conducted to investigate the effects of a NO donor (SNAP) and a NOS inhibitor (LNNA) on the survival rate of INS-1 pancreatic cells. In the first group of experiments, three different doses of the NO donor (SNAP; 50, 100, 200 μM) were used to test the effects of the NO donor on the survival of INS-1 pancreatic cells. As shown in Fig. 1a, SNAP produced concentration- and duration-dependent adverse effects on the survival of these cells. The lowest concentration of SNAP (50 μM) and the shortest duration of exposure (24 h) decreased the survival rate of INS-1 pancreatic cells, significantly. However, the maximum concentration of the SNAP (200 μM) and the longest duration of exposure (72 h) produced the greatest reduction in the survival of the INS-1 pancreatic cells. An intact survival was observed in control cells which did not receive any treatment with SNAP.

On the other hand, the results of the second group of experiments have shown differential responses in the presence of the NO inhibitor, LNNA (Fig. 1b). A significant increase in the survival rate of the INS-1 pancreatic cells was observed in response to a low concentration of LNNA (50 μM) for exposure periods of 48 and 72 h. The same pattern was reported in response to an exposure period of 72 h and when the cells were treated with two higher concentrations of LNNA (100, 200 μM). However, these two concentrations produced a reduction in the survival rate of the cells at exposure time of 24 h. No changes in the survival rate were observed in control cells which did not receive treatment with LNNA.

### Effects of combined treatment with SNAP and a muscarinic acetylcholine receptor antagonist on the survival rate of INS-1 pancreatic cells

**Fig. 2:**
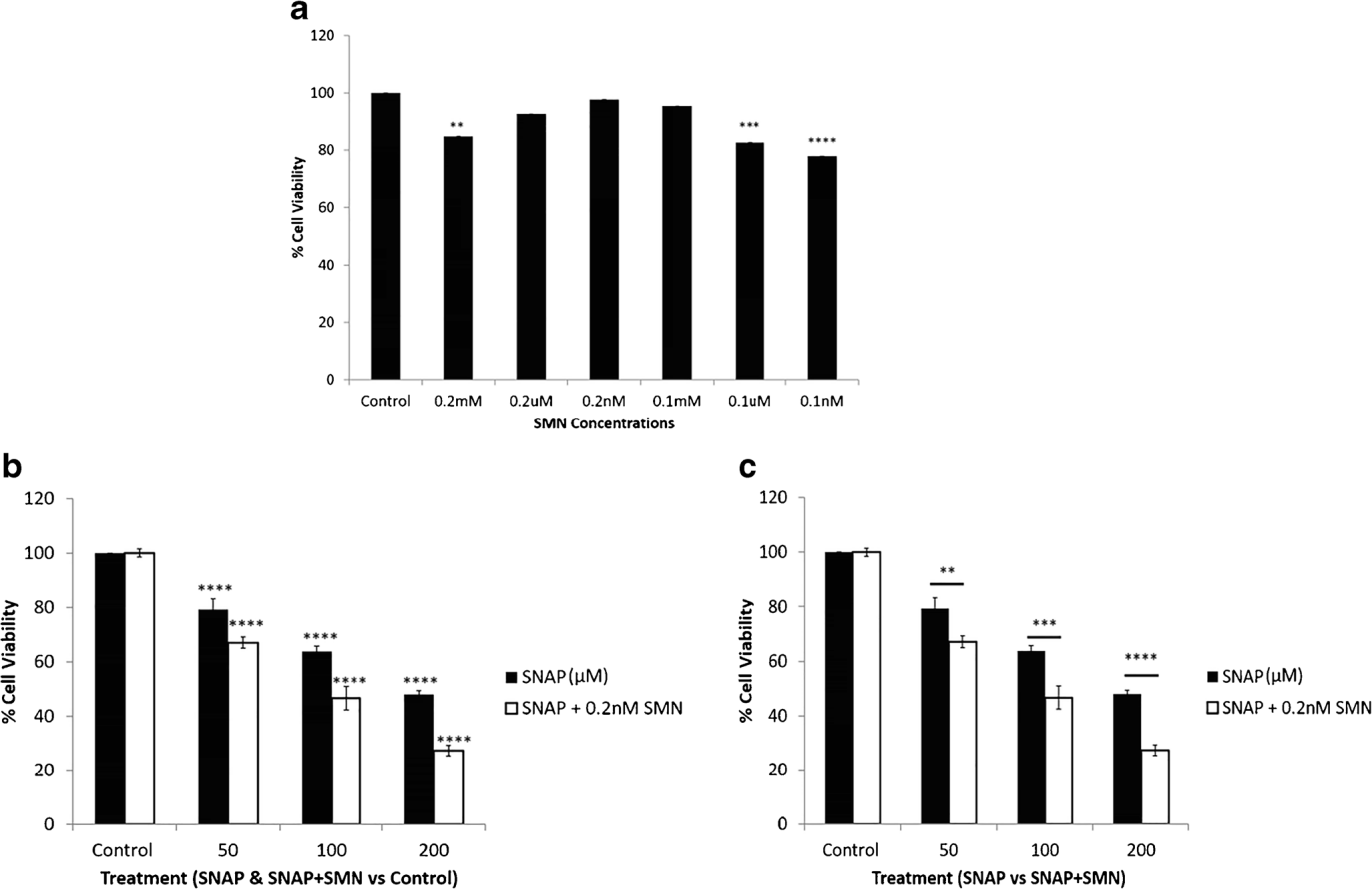
Effects of combined treatment of SNAP and SMN on the survival rate of INS-1 pancreatic cells. **a** Compared to other doses of SMN, 0.2 nM seemed to be the optimal dose for the survival of INS-1 pancreatic cells (*n* = 3, **P < 0.01, ***P < 0.001, ****P < 0.0001). **b** SNAP alone produced concentration-dependent reduction in survival rate compared with controls and SNAP combined SMN decreased the survival rate significantly compared with controls (****P < 0.0001; *P* values refer to differences between effects of SNAP and SNAP combined with SMN compared with controls). **c** SNAP produced a dose-dependent reduction in the survival rate of INS-1 pancreatic cells and this effect was significantly enhanced in the presence of SMN which reduced the survival rate in a concentration-dependent manner (*n* = 3, **P < 0.01, ***P < 0.001, ****P < 0.0001). *P* values refer to differences between the effects of SNAP alone and SNAP with SMN on the survival rate of INS-1 pancreatic cells. All values are expressed as ± SE. Abbreviations: INS-1, insulin-producing pancreatic cells; SMN, scopolamine methyl nitrate; SNAP, *S*-nitroso-*N*-acetylpenicillamine

Another group of experiments was conducted in order to investigate the effects of combined treatment with SNAP and a muscarinic acetylcholine receptor antagonist (mAChR; SMN) on the survival rate of the INS-1 pancreatic cells (Fig. 2). As shown in Fig. 2a, different doses of SMN alone were used to determine the optimal dose to investigate the effects of cholinergic blockade on survival rate of INS-1 pancreatic cells and it was found that a dose of 0.2 nM produced no change in the survival rate. This dose was used for all further investigations.

As shown in Fig. 2b, SNAP alone produced a concentration-dependent reduction in survival rate compared with controls and SNAP combined with SMN decreased the survival rate significantly compared with controls. SNAP produced a dose-dependent reduction in the survival rate of INS-1 pancreatic cells, and this effect was significantly enhanced in the presence of SMN. The latter produced a significant reduction in the survival rate, and this effect was concentration-dependent (Fig. 2c).

### Effects of combined treatment with SNAP and a muscarinic receptor blocker on insulin secretion

**Fig. 3:**
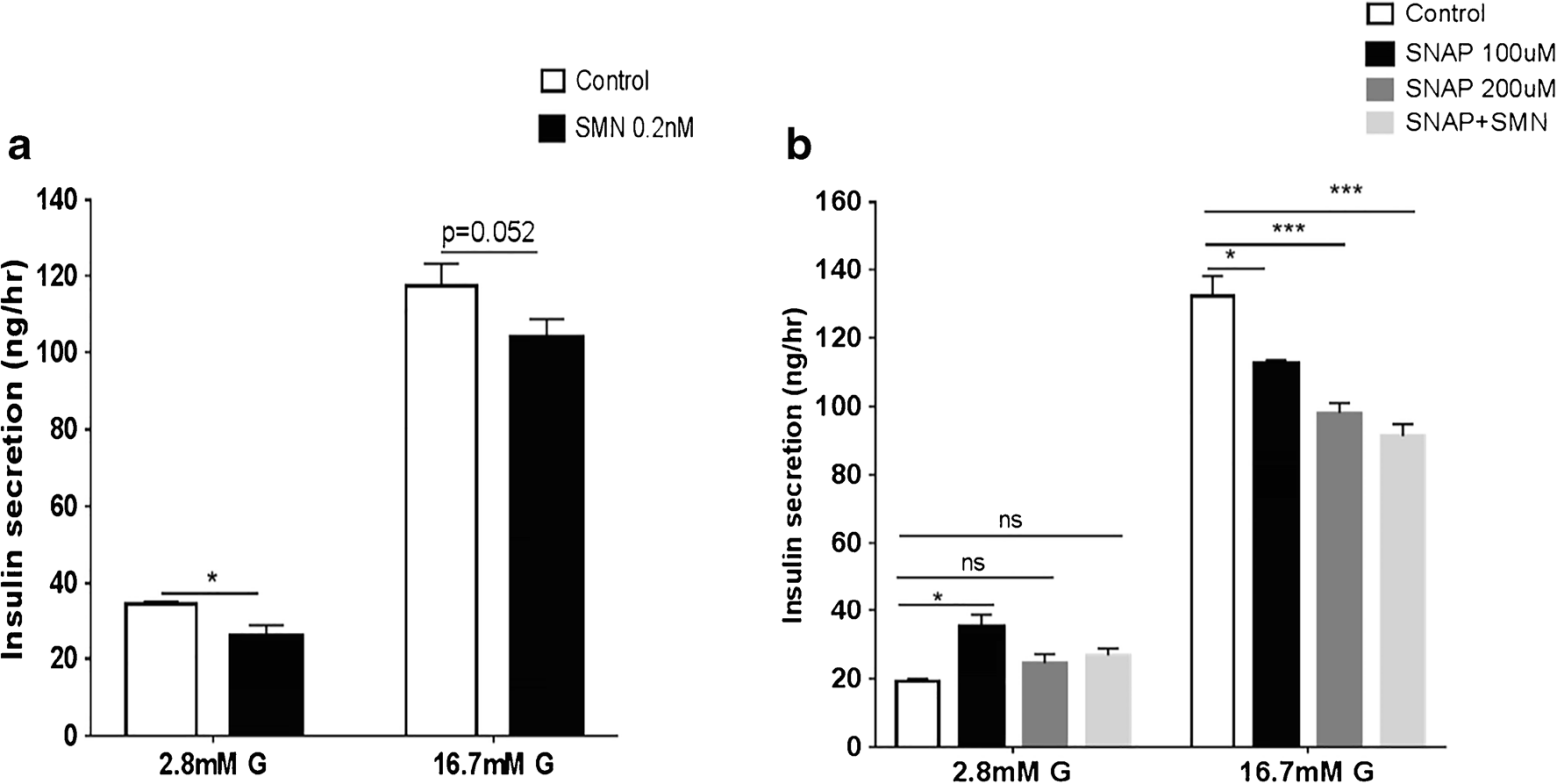
Effects of combined treatment with SNAP and SMN on insulin secretion. **a** A modest but significant reduction in insulin secretion was observed in response to 2.8 mM, but not 16.7 mM in the presence of SMN. **b** SNAP produced a concentration-dependent reduction in glucose-induced insulin secretion and these effects were enhanced significantly in the presence of SMN (*n* = 3, *P < 0.05, ***P < 0.001). All values are expressed as ± SE. Abbreviations: NS, not significant; SMN, scopolamine methyl nitrate; SNAP, *S*-nitroso-*N*-acetylpenicillamine

The effects of SMN, a mAChR antagonist, on glucose-induced insulin secretion were investigated. Two concentrations of glucose (2.8 mM and 16.7 mM) were used to stimulate insulin secretion, and, as shown in Fig. 3a, SMN produced a significant reduction in 2.8 mM glucose-induced insulin secretion while the response to 16.7 mM glucose was not statistically significant. Two concentrations of SNAP, 100 μM and 200 μM, reduced insulin release, which was stimulated by 16.7 mM glucose, significantly. This reduction was concentration-dependent and was significantly enhanced in the presence of mAChR blockade (Fig. 3b). On the other hand, SNAP with concentration of 100 μM produced an increase in insulin secretion that was stimulated by low concentrations of glucose (2.8 mM).

### Effects of cytokines on the survival rate of the INS-1 pancreatic cells and insulin secretion

**Fig. 4:**
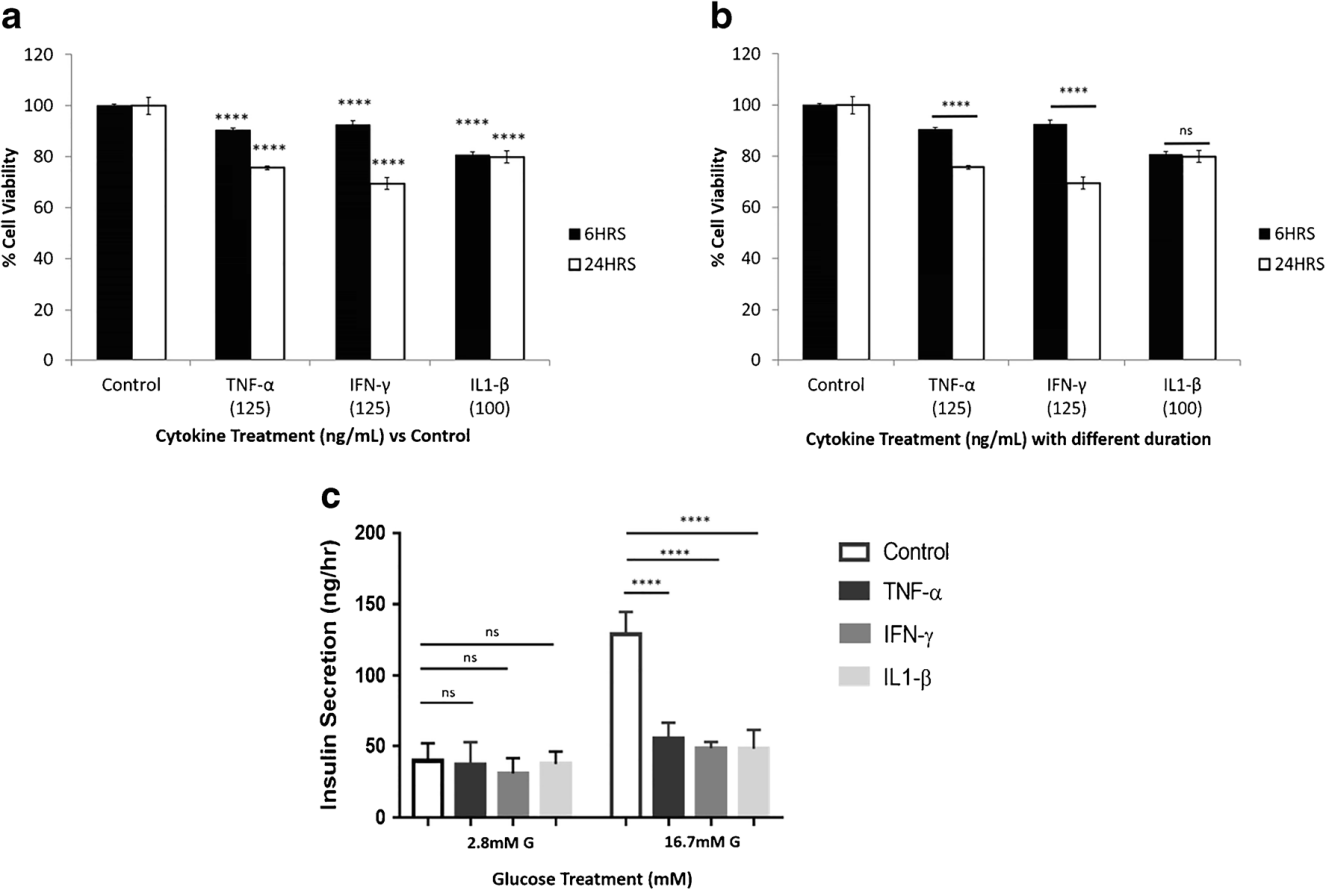
Effects of cytokines on the survival rate of the INS-1 pancreatic cells and glucose-induced insulin secretion. **a** TNF-α, IFN-γ, and IL1-β reduced the survival rate of the INS-1 pancreatic cells compared with control conditions (*n* = 3, ****P < 0.0001). **b** Treatment of INS-1 pancreatic cells with TNF-α and IFN-γ for longer duration (24 h) produced more significant reduction in the survival rate compared with 6-h treatment (*n* = 3, ****P < 0.0001). **c** A significant reduction in insulin secretion was observed in the presence of the three cytokine, and this effect was most pronounced in response to the highest concentration of glucose (*n* = 3, ****P < 0.0001). All values are expressed as ± SE. Abbreviations: IFN-γ, interferon-γ; IL1-β, interleukin 1β; NS, not significant; TNF-α, tumor necrosis factor-α

The effects of three cytokines (TNF-α, IFN-γ, IL1-β) on the survival rate of INS-1 pancreatic cells were examined. A significant reduction in the survival of these cells in response to the presence of TNF-α, IFN-γ, and IL1-β for a duration of 6 and 24 h was noted compared with controls (Fig. 4a). As shown in Fig. 4b, the treatment of INS-1 pancreatic cells with TNF-α, IFN-γ for a longer duration (24 h) produced a greater reduction in the survival rate compared with 6-h treatment. The effects of these cytokines on glucose-induced insulin secretion were also assessed, and as shown in Fig. 4c, TNF-α, IFN-γ, and IL1-β produced a pronounced reduction in glucose-stimulated insulin secretion, while no significant differences were observed at basal (2.8 mM glucose) insulin secretion.

### Assessment of NO production and gene expression of NOS_3_ in the presence of cytokines

**Fig. 5:**
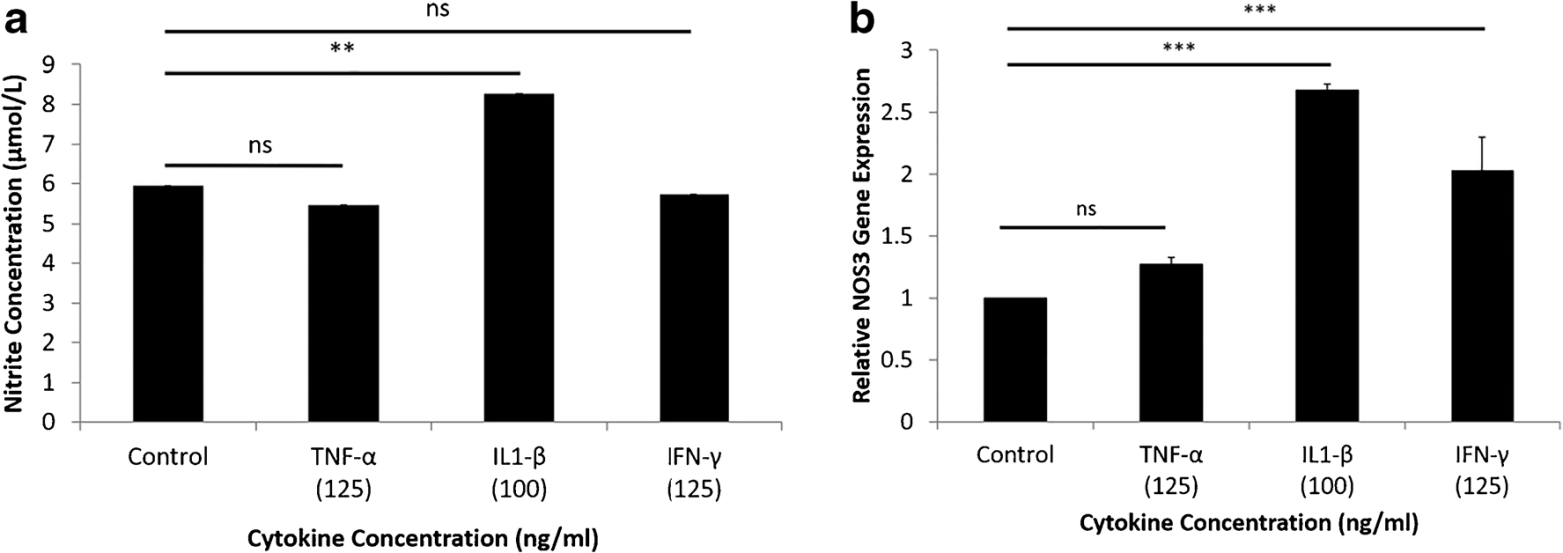
Production of NO and NOS_3_ expression in the presence of cytokines. **a** A significant increase in production of NO was noted in the presence of IL1-β, compared with other cytokines. **b** Exposure to IL1-β and IFN-γ produced a significant increase in NOS_3_ expression, whereas IFN-α had no effect on the expression of NOS_3_ (*n* = 3, **P < 0.01, ***P < 0.001). All values are expressed as ± SE. Abbreviations: IFN-γ, interferon-γ; IL1-β, interleukin 1β; NS, not significant; TNF-α, tumor necrosis factor-α

As shown in Fig. 5, the production of NO was investigated in the absence and presence of cytokines (TNF-α, IFN-γ, IL1-β). The production of NO was significantly increased in the presence of IL1-β. However, no change in the production of NO in the presence of the other cytokines (TNF-α, IFN-γ) was noted (Fig. 5a). A significant increase in NOS_3_ expression was noted in the presence of IL1-β and IFN-γ. However, the presence of TNF-α did not change NOS_3_ expression (Fig. 5b).

### Expression of NOS_3_ in the presence of a NO donor and a muscarinic receptor blocker

**Fig. 6:**
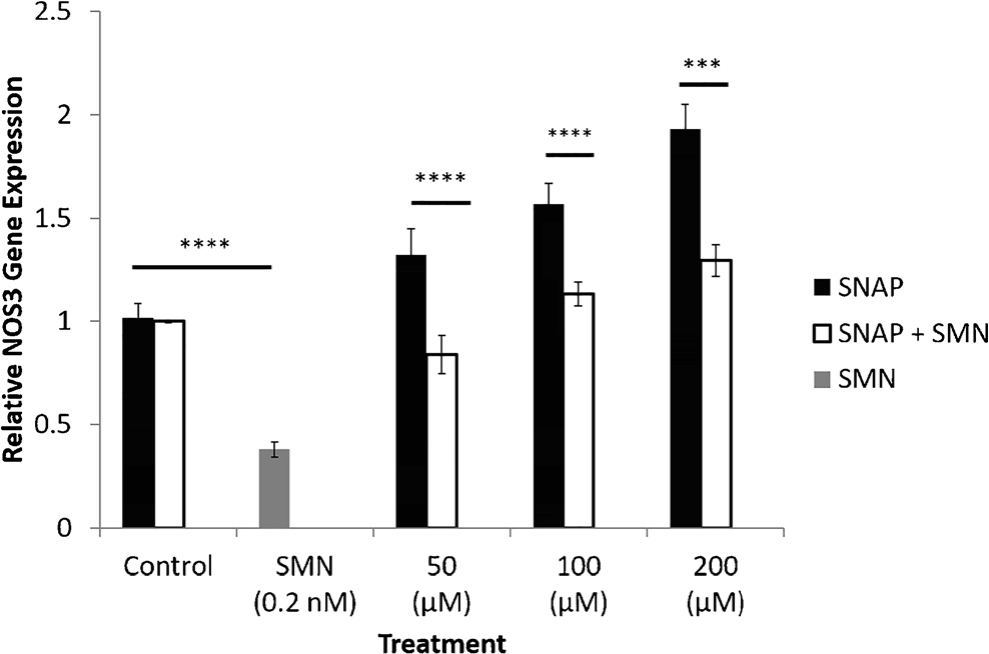
NOS_3_ expression in the presence of a NO donor and a muscarinic receptor antagonist. NOS_3_ expression was increased and decreased in response to SNAP and SMN, respectively. Combined treatment with SNAP and SMN produced a significant reduction in the excitatory effects SNAP on NOS_3_ expression (*n* = 3, ***P < 0.001, ****P < 0.0001). All values are expressed as ± SE. Abbreviations: INS-1, insulin-producing pancreatic cells; NO, nitric oxide; NOS_3_, nitric oxide synthase; SMN, scopolamine methyl nitrate; SNAP, *S*-nitroso-*N*-acetylpenicillamine

A separate group of experiments was conducted to examine the effects of a NO donor and a muscarinic receptor antagonist on NOS_3_ expression. Although SNAP produced a significant and concentration-dependent increase in NOS_3_ expression, SMN attenuated this expression significantly. In addition, a pronounced reduction in expression of NOS_3_ was observed in response to combined treatment with SNAP and SMN (Fig. 6).

### Gene expression of cholinergic receptors in the presence of a NO donor and a muscarinic receptor antagonist

**Fig. 7:**
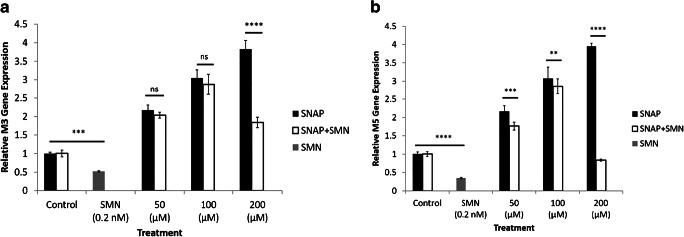
Expression of cholinergic receptors in the presence of a NO donor and a muscarinic receptor antagonist. Expression of M_3_ and M_5_ receptors was reduced significantly in response to SMN. **a** SNAP (200 μM) alone increased the expression of M_3_ receptors, significantly. **b** SNAP alone (50, 100, and 200 μM) increased the expression of M_5_ receptors, significantly. SMN along with SNAP decreased the stimulatory effects of SNAP on the expression of the two receptors (*n* = 3, **P < 0.01, ***P < 0.001, ****P < 0.0001). All values are expressed as ± SE. Abbreviations: M_3_, muscarinic acetylcholine receptor 3; M_5_, muscarinic acetylcholine receptor 5; NS, not significant; SMN, scopolamine methyl nitrate; SNAP, *S*-nitroso-*N*-acetylpenicillamine

Additional experiments examined the expression of cholinergic receptors in the presence of a NO donor and a muscarinic receptor antagonist. SMN alone produced a significant reduction in the expression of M_3_ and M_5_ receptors as shown in Fig. 7 a and b, respectively. In addition, SNAP alone resulted in a significant increase in the expression of M_3_ (SNAP; 200 μM) and M_5_ (SNAP; 50, 100, and 200 μM). These effects were attenuated significantly in response to combined treatment with SNAP and SMN.

### Protein expression of cholinergic receptors in the presence of NO donor and cholinergic blocker

**Fig. 8:**
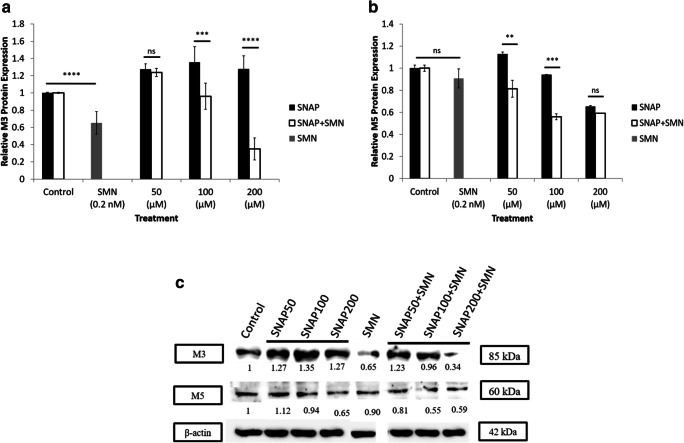
Protein expression of M_3_ and M_5_ receptors in the presence of SNAP and SMN. **a** Protein expression of M_3_ receptor was sensitive to SMN, and a significant reduction was reported in the expression of M_3_ receptors. Combination of SNAP (100 and 200 μM) and SMN attenuated the expression of M_3_ receptors in a dose-dependent fashion (*n* = 3, ***P < 0.001, ****P < 0.0001). **b** No change was observed in the expression of M_5_ in response to SMN. Combination of SNAP (50 and 100 μM) and SMN reduced the expression of M_5_ significantly; however, the highest dose of SNAP (200 μM) did not affect the expression of M_5_ (**P < 0.01, ***P < 0.001). **c** Representative western blot of M_3_ and M_5_ in response to the presence of SMN and different concentrations of SNAP; protein levels were corrected to actin expression. All values are expressed as ± SE. Abbreviations: M_3_, muscarinic acetylcholine receptor 3; M_5,_ muscarinic acetylcholine receptor 5; NS, not significant; SMN, scopolamine methyl nitrate; SNAP, *S*-nitroso-*N*-acetylpenicillamine

Protein expression of M_3_ and M_5_ receptors was assessed in the presence of SNAP alone, SMN alone, and in combination of both. The expression of the two receptors was sensitive to SMN; however, more significant outcomes were reported in the expression of M_3_. As shown in Fig. 8a, SMN reduced the expression of the M_3_ significantly and the combination of SNAP and SMN attenuated the expression of M_3_ receptor in a concentration-dependent manner. Figure 8b shows a slight change in the expression of M_5_ receptor; however, the combination of SNAP (two concentrations; 50 and 100 μM) and SMN produced significant reduction in the expression of the M_5_ receptor and no significant changes were observed in response to the highest concentration of SNAP (200 μM). Representative western blot of M_3_ and M_5_in response to the presense of SMN and different concentrations of SNAP were shown in Fig. 8c.

## Discussion

It is well-documented that progressive impairment of pancreatic β cells and associated dysfunction of insulin secretion are the original signatures of DM. Although previous studies have attempted to determine the participating elements in β cell pathogenesis, the precise mechanisms are still poorly understood [6].

Previously, we and others have shown that insulin secretion seems to be modulated by brainstem cholinergic pathways and NO. These in vivo experiments have confirmed that the blockade of cholinergic pathways and the inhibition of NO synthesis had inhibitory and excitatory effects on insulin secretion, respectively [24].

Cytokines have been implicated as strong candidates for dysfunction of β cells and, subsequently, the secretion of insulin. It is believed that cytokines, including IL-1β, TNF-α, and IFN-γ, mediate this effect via induction of NO formation. It seems that the latter is involved as a causative agent of most of the inhibitory and cytotoxic effect of the cytokines on the pancreatic islets [12, 36].

The present study is the first to find that there is an interaction between the nitrergic and cholinergic pathways to modulate insulin secretion from the β cells. Interestingly, it was also found that the source of NO is cytokine-dependent and the muscarinic acetylcholine receptors are the mediators of the cholinergic involvement in regulation of insulin secretion.

In agreement with the present results, previous reports have shown that SNAP produced adverse effects on the survival of pancreatic β cells [21]. SNAP produced both apoptosis and necrosis in the pancreatic β cells, and it seems that the main pathological mechanism is the disruption of the mitochondrial membrane potential [21]. Although the impact of SNAP on pancreatic islets has been studied previously, the present study provides greater depth by examining a range of concentrations and exposure durations to SNAP using INS-1 cells. Although SNAP has a half-life of several hours, the effects of the released NO have been documented for days. To further confirm the effects of SNAP on survival rate of pancreatic cells, control experiments were conducted using similar conditions and timelines but in the absence of SNAP [30, 37].

In addition, the present study investigated the effects of NOS inhibitors, using the same range of concentrations and exposures, on the viability of the pancreatic β cells. It was evident that the NO donor produced adverse effects on the survival rate of the pancreatic β cells, whereas the viability of these cells was decreased in response to the presence of NOS inhibitors. Although the effect of the latter was not the focus of the present study, an interesting finding was noted; the positive impact of LNNA on the survival rate of the β cells was only observed after a long duration of exposure. Given that the RPMI media used in the present study contains arginine, a NO inhibitor, may compete with it to produce an increase in the survival of β cells. Therefore, LNNA took a longer time to exhibit a positive impact. In addition, it has been found that β cells constitutively express nNOS and eNOS and this may explain the observed effects of LNNA on the survival of the β cells [3]. The basal mechanism of NO generation was not the focus of the present study. Therefore, the appropriate control for this experiment was the assessment of cell viability in the absence of LNNA. However, it will be of great interest to conduct further studies to draw a firm conclusion about the proliferative effects of LNNA on β cells.

Interestingly, the inhibitory effects of SNAP on the survival rate were enhanced by the presence of a muscarinic antagonist suggesting an interaction between these two agents in modulating the survival of β cells. A recent study by Gupta et al. has shown that ACh receptors play a vital role in maintaining the mass and survival of pancreatic β cells. Similar to the present study, Gupta and his group used the INS-1 832/13 pancreatic β cell line. Therefore, it provides strong support and validation to our present results [14].

It is well established that ACh is a neurotransmitter that is involved in the regulation of secretory function of the insulin secreting pancreatic β cell [32]. However, the exact interactive mechanism between cholinergic and nitrergic inputs is yet to be defined. In line with other studies, the present study has demonstrated that the blockade of ACh receptors attenuated glucose-induced insulin secretion [23]. Previously, we and others have used atropine as a blocker of cholinoceptors but since this agent was not available, SMN, which is also a non-selective muscarinic antagonist, was used. Cholinergic blockade by SMN enhanced the inhibitory effects of SNAP on glucose-induced insulin secretion emphasizing an interrelationship between nitrergic and cholinergic mechanisms. Previous reports have suggested a coupling mechanism between muscarinic receptors and NOS activity in regulation of secretion of amylase from salivary glands. Similarly, the present study provides novel insights about the coupling of these two systems in regulation of insulin secretion [33]. On the other hand, it was found that insulin secretion from the β cells was increased in response to a low concentration of glucose after treatment with SNAP 100 μM. It seemed that SNAP not only impaired glucose stimulated insulin secretion but also elevated basal insulin release, significantly. Although the underlying mechanism of the elevated basal insulin secretion is unknown, it might be related to glucose sensing or disruption of membrane rafts [4, 18, 26]. Recently, various other studies reported similar elevation in basal secretion following palmitate treatment [17, 27].

The negative impact of cytokines on the function and the survival of pancreatic β cells have been demonstrated, and this further supported by our current findings [22, 31]. We have investigated the effects of different cytokines on survival and function of β cells, and both TNF-α and IFN-γ reduced the survival of these cells, significantly. In addition, TNF-α, IFN-γ, and IL1β produced a pronounced reduction in insulin secretion. Interestingly, the production of NO and expression of NOS_3_ were both increased in the presence of cytokines, mainly IL1β, suggesting that the adverse effects of IL1β are mediated via induction of NO production. It is noteworthy that the production of NO and expression of NOS_3_ were not significant in response to TNF-α. Previous studies have demonstrated that the latter alone does not affect NO production significantly. However, in the presence of IFN-γ, large amounts of NO were produced by IL1-β [19]. In agreement with this, a study by Chambers et al. has demonstrated that cytokine-induced β cell death is primarily mediated by production of NO [11]. In agreement with our findings, this previous study has also showed that the effects of cytokines were mainly attributed to IL1 [11].

Recently, several biochemical and molecular studies have proposed various mechanisms to explain the inhibitory effects of NO on the survival and secretory function of β cells. This includes targeting iron-sulfur–containing enzymes [35], reductions in cellular levels of ATP [15], and induction of endoplasmic reticulum stress and prolonged unfolded protein response activation [9, 11]. On the other hand, the present study investigated the importance of the ACh receptors in modulating the effects of NO on β cell survival and insulin secretion. The blockade of mAChRs enhanced the inhibitory effects of NO donor on the survival of β cells and the secretion of insulin suggesting an interrelationship between these two systems.

The expression of NOS_3_ was increased in response to the presence of a NO donor, and this effect was concentration-dependent suggesting that the inhibitory effects of NO donor on survival and secretory function of β cells may be partially mediated by increased levels of NOS_3_. Although the involvement of different NOS isoforms in regulation of insulin secretion is controversial, several reports have demonstrated that NOS_3_ and NOS_1_ are constitutively expressed in pancreatic vasculature and neurons, respectively [7]. Compared with other isoforms, NOS_3_ seems to play a major role in modulation of pancreatic secretion and has been detected in rat and human pancreatic islets; hence, the present study focused on the involvement of NOS_3_ in this process [7].

In contrast, mAChR blockade alone attenuated the expression of NOS_3_ and the excitatory effects of the NO donor on expression of NOS_3_ suggesting that the inhibitory effects of ACh receptor blockade can override the excitatory effects of NO on expression of NOS_3_. Therefore, further experimentation was conducted to investigate the nature of this interaction.

Not surprisingly, a mAChR antagonist decreased the expression of M_3_ and M_5_ mAChRs significantly, and this may explain the mechanism of action by which these agents modulate insulin secretion. It is noteworthy that protein expression analysis showed that M_3_ mAChR protein expression was more sensitive to the mAChR blockade compared with M_5_ expression. The involvement of M_3_ and M_5_ mAChRs is supported by previous reports that stimulation of insulin secretion is mediated by activation of M_3_ and M_5_ mAChRs in different β cell lines including rodents and human [23, 28].

NO donor seems to increase the expression of M_3_ and M_5_ mAChRs, and this effect was attenuated in the presence of mAChR antagonist. Although the coupling relationship between NOS and mAChR has been reported previously for regulation of amylase secretion from the salivary gland, the present study is the first to demonstrate this phenomenon in modulation of insulin secretion [33]. Several mechanistic possibilities have been considered to explain the cross-talk between the nitrergic and cholinergic pathways. This may include protein kinase C, a core element in the signaling pathways of the mAChRs, and which is involved in regulation of NOS activity [20]. In addition, Ca^2+^ mobilization which is activated by muscarinic receptors plays an important role in NO signaling pathways [20]. Furthermore, the presence of both nitrergic and cholinergic neurotransmission in the pancreatic tissue suggests an interaction between the neurotransmitters of these two systems to modulate insulin secretion [25].

On the other hand, one can argue that the NO donor had adverse effects on insulin secretion and the survival of β cells and yet an increase in the expression of M_3_ and M_5_ mAChRs was reported in the presence of the NO donor. However, two key factors were considered to explain this controversy: (i) NO has a dual role in regulation of β cell survival and insulin secretion; both negative and positive effects and anti- and pro-apoptotic activities have been reported based on NOS isoforms and NO concentrations, and (ii) the interaction between the cholinergic and nitrergic systems seems to be dependent on the nature of the pancreatic tissues, and some of the coupling mechanisms between these two systems are confined to neuronal pancreatic tissue [2, 20].

## Conclusion

The findings of the present study have shown that a NO donor, muscarinic acetylcholine receptor blockade, and cytokines produced adverse effects on survival rate of β cells and insulin secretion. The effect of cytokines seems to be mediated via production of NO, and this was evident by the increase in NO production and gene expression of NOS_3_ in response to cytokines. In addition, this study has demonstrated an interaction between the mAChRs and NO in modulation of the survival of β cells and insulin secretion. Although the present findings are interesting, further studies need to be conducted to investigate the involvement of different muscarinic receptor subtypes, and therefore, selective cholinergic blockers will be used. Moreover, future studies will test the effects of muscarinic agonists and, if they are able to reverse the impairments that are caused by SNAP.

## Abbreviations

Ach: Acetylcholine
NO: Nitric oxide
DM: Diabetes mellitus
SNAP: *S*-nitroso-*N*-acetylpenicillamine
LNNA: L-NG-nitro-l-arginine
NOS_3_: Nitric oxide synthase 3
M_3_: Muscarinic acetylcholine receptor 3
M_5_: Muscarinic acetylcholine receptor 5
mAChR: Muscarinic acetylcholine receptor antagonist
IFN-γ: Interferon-γ
IL1-β: Interleukin-1β
INS-1: Insulin-producing pancreatic cells
TNF-α: Tumor necrosis factor-α
SMN: Scopolamine methyl nitrate
